# Coexistence of atherosclerosis and fistula as a cause of angina pectoris: a case report

**DOI:** 10.1186/1757-1626-3-70

**Published:** 2010-02-23

**Authors:** Dimitris P Papadopoulos, Christos V Bourantas, Chrisostomos K Ekonomou, Vasilios Votteas

**Affiliations:** 1 Department of Cardiology, Laiko Hospital, 39 Karneadou Street, Athens, 10675, Greece

## Abstract

**Introduction:**

Coronary artery fistulas are abnormal communications between a coronary artery and a cardiac chamber or a major vessel (vena cava, pulmonary vein, pulmonary artery). They are usually diagnosed by coronary arteriography. Clinical presentations are variable depending on the type of fistula, shunt volume, site of the shunt, and presence of other cardiac conditions.

**Case presentation:**

This report describes a 46-year-old Greek female patient who was admitted to the hospital because of an acute coronary syndrome. She underwent coronary angiogram which showed a coronary artery fistula from the left anterior descending artery to the main pulmonary artery and severe coronary disease. The patient was referred for coronary artery bypass surgery and fistula closure operation.

**Conclusions:**

Coronary artery fistulas between left anterior descending artery and main pulmonary artery are very rare anomalies. This case report describes a patient with this anomaly combined with severe coronary disease, reviews the current literature and discusses the available options for treating this rare condition.

## Introduction

Coronary artery fistulas (CAF) are rare congenital or acquired coronary artery anomalies that can originate from any of the three major coronary arteries and drain in all the cardiac chambers and great vessels. Their incidence in the overall population is estimated to be about 0.002% and constitute 0.13% of the congenital cardiac lesions [1]. The majority of these fistulas drain into the systemic venous side of the circulation. Drainage of the fistula into the pulmonary trunk has been reported in 17% of cases [2]. To the best of our knowledge, a connection between left anterior descending artery (LAD) and main pulmonary artery has been reported in the literature very rare, as a case.

## Case presentation

A 46-year-old Greek female (weight 60 Kg, height 1.65 cm) with symptomatic stable angina and myocardial ischemia documented at 99mTc-MIBI SPECT scintigraphy admitted to the hospital complaining of retrosternal chest pain appeared at rest one hour before admission. The patient was also known to suffer from hypertension and hypercholesterolemia and had a strong family history of ischemic heart disease as her father and her brother had a myocardial infarction at the age of 55 and 45 respectively. She was taking aspirin, b-blocker and statin prior to this admission.

**Figure 1:**
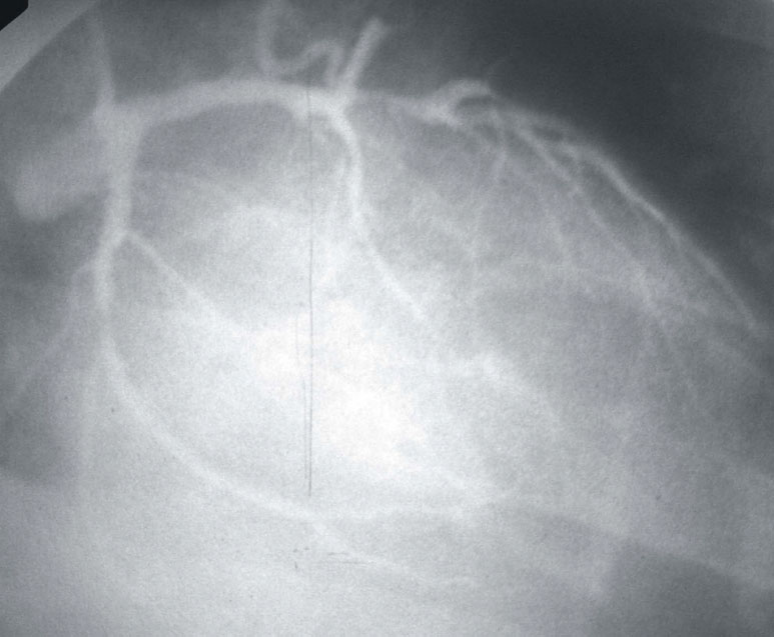
**Fistula connection between LAD and main pulmonary artery**.

Clinical examination demonstrated normal heart and chest sounds, an elevated blood pressure 155/90 mmHg and a heart rate 85 beats per minute. The jugular venous pressure was not elevated while the electrocardiogram revealed sinus rhythm with ST depression in the precordial leads V2 to V6. There was no evidence of heart failure. The patients was put on fondaparinux, agrastat, clopidogrel 300 mg, nitrates and her aspirin b-blocker and statin were continued. She underwent a coronary arteriogram next day which revealed a CAF from LAD to the main pulmonary artery, a blocked right coronary artery and significant stenoses on the other vessels (Figure 1). The patient was admitted for coronary artery bypass surgery and fistula closure with multiple stitches. After the operation the patient was asymptomatic and a nuclear scan, performed 6 months later, showed a normal myocardial perfusion.

## Discussion

CAF are rare malformations. In a review of 363 cases with CAF, 50% of the fistulas were found to arise from the right coronary artery, 42% from the left coronary artery, and 5% from both coronary arteries. The most common site of drainage was the right ventricle (41%), followed by the right atrium (26%) and the pulmonary artery (17%) [3]. Approximately, 56% of the bilateral fistulas and only 17% of the unilateral fistulas were found to drain into the pulmonary artery. A fistula between the left main coronary and the pulmonary artery is a rare finding [4]. Multiple coronary-pulmonary artery fistulas have also been reported. Abhyankar et al. reported a case of a patient who had non-exertional chest pain and a normal nuclear stress test. Coronary arteriography demonstrated no obstructive coronary lesions and multiple coronary artery fistulas which originated form all the three arteries (3 from the LAD, 2 from the left circumflex and 2 from the right coronary artery) and drained into the main pulmonary artery [5].

Patients with CAF may be asymptomatic or develop symptoms of pulmonary congestion due to left to right shunt. Complications such as bacterial endocarditis, thrombosis, distal embolization, aneurysm formation, dissection, rupture, premature atherosclerosis, pulmonary hypertension, myocardial ischemia, or infarction, have also been reported [6,7].

Management of CAF remains controversial since opinions vary on which procedure should be performed to these patients. Liberthson et al. reviewed 187 cases, and found low operative mortality (1%) and incidence of complications (7%) in young patients (<20 years old) [6]. On the other hand the postoperative mortality was higher in patients older than 20 years old (7%). In these patients the risk of postoperative myocardial infarction (7%) and other complications (23%) was also increased. Other large series recommend surgery in all cases of CAF in childhood irrespective of symptoms or size of the shunt [8,9]. In addition, Onorati et al. reported that patients undergoing surgical treatment of cardiac disease, associated CAF should always be treated [10]. Although it is safer, in patients with giant CAF, to patch the outflow of CAF from the outflow chamber, in the majority of cases CAF should be identified intraoperatively and closed with multiple running stitches [9].

The increased experience and the improved devices and techniques provide a variety of treatment options. The management of each individual patient who has CAF should depend on the anatomy of the fistula, the presence or absence of associated defects, and the experience of the interventional cardiologists and surgeons. In our case surgical treatment (coronary bypass surgery and CAF closure) was preferred for treating this patient as there was a chronic total occlusion in the right coronary artery which was not suitable for percutaneous coronary intervention.

## Consent

Written informed consent was obtained from the patient for publication of this case report and accompanying images. A copy of the written consent is available for review from the journal's Editor-in-Chief.

## Competing interests

The authors declare that they have no competing interests.

## Authors' contributions

All authors read and approved the final manuscript. DPP assessed the patient in the acute assessment unit and wrote the manuscript. CVB analyzed and interpreted the patient data and was a major contributor in writing the manuscript. CKE and VV were also contributed to the writing of the manuscript. In addition CKE performed the coronary angiography, arranged patient further management and reviewed the literature while VV is following up the patient in the outpatient clinic.
